# Oncologic Safety of Immediate Oncoplastic Surgery Compared with Standard Breast-Conserving Surgery for Patients with Invasive Lobular Carcinoma

**DOI:** 10.1245/s10434-024-15326-5

**Published:** 2024-05-07

**Authors:** Israel Falade, Kayla Switalla, Astrid Quirarte, Molly Baxter, Daniel Soroudi, Harriet Rothschild, Shoko Emily Abe, Karen Goodwin, Merisa Piper, Jasmine Wong, Robert Foster, Rita A. Mukhtar

**Affiliations:** 1 School of Medicine, University of California–San Francisco, San Francisco, CA USA; 2 University of Minnesota Medical School, Minneapolis, MN USA; 3 Division of Surgical Oncology, Department of Surgery, University of California–San Francisco, San Francisco, CA USA; 4 Division of Plastic and Reconstructive Surgery, Department of Surgery, University of California–San Francisco, San Francisco, CA USA

**Keywords:** Oncoplastic Reduction Mammoplasty, Invasive Lobular Carcinoma, Breast Conserving Surgery, Positive Margins, Recurrence Free Survival, Lumpectomy, Oncologic Safety, Surgical Outcomes

## Abstract

**Background:**

Invasive lobular carcinoma (ILC) of the breast grows in a diffuse pattern, resulting in a high risk of positive margins at surgical resection. Oncoplastic approaches have been shown to reduce this risk, but concerns persist around the safety of immediate oncoplastic surgery for those with ILC. This study evaluated the short- and long-term oncologic outcomes of immediate oncoplastic surgery for patients with ILC.

**Methods:**

This study retrospectively analyzed an institutional database of stages I to III ILC patients who underwent breast-conserving surgery (BCS) with or without immediate oncoplastic surgery (oncoplastic closure or oncoplastic reduction mammoplasty [ORM]). The study compared positive margin rates, rates of successful BCS, and recurrence-free survival (RFS) by type of surgery.

**Results:**

For 494 patients the findings showed that the use of immediate ORM was associated with significantly lower odds of positive margins (odds ratio [OR], 0.34; 95 % confidence interval [CI], 0.17–0.66; *p* = 0.002). Both lumpectomy with oncoplastic closure and ORM were significantly associated with higher rates of successful BCS than standard lumpectomy (94.2 %, 87.8 %, and 73.9 %, respectively; *p* < 0.001). No difference in RFS was observed between those undergoing immediate oncoplastic surgery and those undergoing standard lumpectomy alone.

**Conclusions:**

The patients with stages I to III ILC who underwent immediate oncoplastic surgery had significant benefits including lower odds of positive margins and higher rates of successful BCS, with both types of immediate oncoplastic surgery showing similar RFS compared with lumpectomy alone. This supports the oncologic safety of immediate oncoplastic surgery for diffusely growing tumors such as ILC, providing it an ideal option for patients desiring BCS.

Invasive lobular carcinoma (ILC), the second most common histologic type of breast cancer, is characterized by a proliferation of carcinoma cells lacking the adhesion protein E-cadherin.^1^ These tumors typically grow in a diffuse pattern, making clinical and radiologic detection of ILC more difficult than for other tumor types.^2^ Consequently, larger tumors and more nodal involvement are diagnosed for patients with ILC than for those with invasive ductal carcinoma (IDC).^3^ This higher stage of disease at presentation can complicate both systemic and local therapy for those with ILC.

Although most ILC tumors are molecularly low risk, presentation with tumors at a higher stage confers high clinical risk, with the resulting discordance making systemic treatment selection more challenging.^4,5^ Additionally, patients with ILC have worse surgical outcomes, with higher rates of positive margins, completion mastectomies, and axillary dissections, all of which may cause greater surgical morbidity.^6–8^

We previously showed that the use of immediate oncoplastic surgery was associated with a significant reduction in the risk of positive margins in a single institutional cohort of patients with stages I to III ILC.^9^ Similarly, several other investigators have shown that oncoplastic approaches, including both oncoplastic closure with minimal tissue rearrangement and more extensive volume reduction techniques such as oncoplastic reduction mammoplasty (ORM), reduce positive margin rates compared with standard breast-conserving surgery alone for patients with breast cancer in general.^10–14^ Due to the well-known increased risk of positive margins at surgical excision for ILC, some early proponents of oncoplastic surgery specifically highlighted lobular histology as an indication for its use.^15^

Despite this, concerns in the surgical literature persist around applying such approaches to patients with the highest risk of positive margins, including those with ILC.^16–21^ Such concerns stem from the perceived potential difficulty of accurately performing a re-excision in the setting of previous tissue rearrangement, as well as concerns about needing to “un-do” an oncoplastic procedure should positive margins occur.^22^ As such, some institutions have implemented the approach of “delayed” oncoplastic surgery for patients with a high risk of positive margins.^23^ With this approach, patients are recommended to first undergo standard BCS without oncoplastic surgery, followed by an oncoplastic procedure as a subsequent operation once margin status has been evaluated. Because this often is recommended for patients with a high risk of positive margins, those with ILC are more likely to undergo delayed oncoplastic surgery at institutions that implement such approaches.^24^

Consequently, the optimal surgical approach for those with ILC is unclear, with two somewhat contradictory concepts guiding decision-making. On the one hand, oncoplastic surgery reduces the risk of positive margins, which suggests that it should be used for those with a high risk of positive margins. On the other hand, managing positive margins after oncoplastic surgery might result in worse outcomes, particularly with regard to accurate performance of re-excision, suggesting that it should not be used initially for those with a high risk of positive margins.

Although several studies show that oncoplastic surgery results in a recurrence risk similar to that with either standard BCS or mastectomy, data on ILC are limited, with no published data to our knowledge considering the oncologic safety of the immediate oncoplastic approach specifically for those with ILC.^13,25,26^

Given the paucity of data to guide surgical management, we evaluated a cohort of patients with early-stage ILC who underwent either standard BCS or BCS followed by immediate oncoplastic surgery, with the goal of determining the oncologic safety of immediate oncoplastic surgery. We evaluated both short- and long-term outcomes including positive margin rates, completion mastectomy rates, and recurrence-free survival (RFS) for patients with ILC who underwent immediate oncoplastic surgery compared with standard BCS.

## Methods

### Data Collection and Study Population

We retrospectively analyzed consecutive cases from a prospectively maintained institutional ILC database containing treatment and outcomes data for patients with ILC who underwent surgery between 1995 and 2023. This study was approved by the University of California–San Francisco (UCSF) institutional review board. Patients who underwent BCS with or without oncoplastic surgery as their initial operative approach were included for analysis.

Baseline clinicopathologic features were collected, including age at diagnosis, T stage, nodal stage, tumor receptor subtype, tumor grade, tissue resection volume in cm^3^, shave margin use, and type of surgery performed. Tumor receptor subtype was defined by estrogen receptor (ER) status (≥1 % staining on immunohistochemistry [IHC] considered positive), progesterone receptor (PR) status (≥1 % staining on IHC considered positive), and human epidermal growth factor receptor-2 (HER2, assessed by IHC and routine fluorescence *in situ* hybridization). Tissue resection volume at initial resection was approximated as cm^3^ by multiplying the dimensions of the lumpectomy specimen as recorded in pathology reports (medial–lateral length × superior–inferior length × anterior-posterior length).

Patients were categorized into the following three groups by type breast-conserving surgery (BCS) performed: standard BCS, lumpectomy with oncoplastic closure, or oncoplastic reduction mammoplasty (ORM). Standard BCS was defined as lumpectomy/partial mastectomy without oncoplastic surgery, performed by a breast surgeon.

At our institution, oncoplastic approaches are typically dual-surgeon cases, with a breast surgeon performing lumpectomy and a plastic surgeon performing either immediate oncoplastic closure (local tissue rearrangement/volume displacement) or immediate volume reduction (most commonly Wise-pattern ORM). Patient selection for BCS with the oncoplastic approach is determined by discussion with the breast surgeon, plastic surgeon, and patient. In general, patients with tumors involving more than two quadrants of the breast on imaging or physical examination are not considered candidates for BCS. For larger-breasted women with favorable tumor location (i.e., upper quadrants) and up to two quadrants involved, BCS with the oncoplastic approach is offered provided the patients understand the risk of positive margins.^27^

In this study, the surgical procedure performed was determined by review of operative reports. We compared clinicopathologic features by type of BCS performed. Additionally, we compared type of surgery initially performed relative to publication of margin consensus guidelines in 2014.^28^

Our primary aim was to determine whether immediate oncoplastic surgery is associated with worse oncologic outcomes than standard BCS. Specifically, we evaluated three outcomes: positive margin rates, successful BCS rates, and RFS. Positive margins were defined as ink on tumor based on review of margin width on pathology reports. For those with positive margins after ORM specifically, the rate and type of subsequent surgery were analyzed.

Our institutional approach to lumpectomy included wire localization of non-palpable lesions until 2016, after which we transitioned to seed localization. Bracketing lesions larger than 4 cm with two localizing devices is commonly used, but at the discretion of the operating surgeon. Routine intraoperative specimen radiographs are used to confirm lesion retrieval and may guide subsequent margin re-excision. The use of routine shave margins for those with ILC has been recommended at our institution since 2018.^9^ Histologic margin status is assessed postoperatively on fixed paraffin-embedded tissue.

Successful BCS was defined as BCS that did not require completion mastectomy. The number of operations required to achieve successful BCS by initial operation performed was calculated. Recurrence-free survival was defined as patient survival without local or distant breast cancer recurrence, with patients who had no recurrence censored at the date of the last follow up visit.

### Statistical Analysis

Clinicopathologic and demographic features were compared across BCS groups using Pearson’s chi-square test and analysis of variance (ANOVA). Multivariable logistic regression models were developed to assess factors associated with odds of positive margins and successful BCS rates. Recurrence-free survival time was evaluated using the log-rank test and Kaplan–Meier survival analysis to account for differences in follow-up time between BCS groups. A multivariable Cox proportional hazards model, right censored at 10 years, was used to assess hazard ratios (HRs) with 95 % confidence intervals (CIs). The multivariable model included known factors associated with recurrence (tumor size, number of positive nodes, and tumor receptor subtype), and the proportional hazards assumption was tested with the log likelihood ratio test, Schoenfeld residuals, and a log-log plot. Two-tailed *p* values lower than 0.05 were considered statistically significant.

## Results

### Demographics

Of 810 consecutive patients with stages I to III ILC, 494 underwent BCS with or without oncoplastic surgery as their initial operative intervention and comprised the study cohort. Among these 494 patients, the average age at ILC diagnosis was 61.4 years and just more than half had T1 tumors (57.5 % T1, 31.3 % T2, 11.2 % T3). Most tumors were of the ER-positive, PR-positive, and HER2-negative receptor subtype (79.7 %) and grade II (64.6 %) (Table 1).

**Table 1:** Patient characteristics and clinicopathologic features by BCS procedure type

	All	Lumpectomy	Lumpectomy with oncoplastic closure	ORM	P value
	N=494	n=326	n=86	n=82	
Age, years^a^	61.4(12.0)	62.5 (12.4)	60.5(11.3)	58.1 (10.4)	0.01
T stage^b^					< 0.001
1	281 (57.5%)	198 (61.5%)	52(61.2%)	31 (37.8%)	
2	153(31.3%)	96(29.8%)	26 (30.6%)	31 (37.8%)	
3	55 (11.2%)	28 (8.7%)	7 (8.2%)	20 (24.4%)	
Tumor size, cm^b^	2.4(1.9)	2.2(1.7)	2.2(1.7)	3.3 (2.5)	0.001
Lumpectomy volume, cm^3c^	94.0 (106.3)	61.7(50.5)	86.9 (76.8)	189.5(170.1)	< 0.01
Shave margins^d^	285(61.6%)	157 (53.0%)	61 (71.8%)	67 (81.7%)	< 0.01
N stage^b^					0.50
0	356 (72.8%)	237 (73.8%)	66 (76.7%)	53 (64.6%)	
1	91 (18.6%)	56(17.4%)	15 (17.4%)	20 (24.4%)	
2	25 (5.1%)	15 (4.7%)	4 (4.7%)	6 (7.3%)	
3	17(3.5%)	13 (4.1%)	1(1.2%)	3 (3.7%)	
Tumor grade^c^					0.25
1	149(30.8%)	108(34.1%)	20 (23.5%)	21 (25.9%)	
2	312(64.6%)	195(61.5%)	62 (72.9%)	55 (67.9%)	
3	22(4.6%)	14 (4.4%)	3 (3.5%)	5 (6.2%)	
Tumor receptor subtype^f^					0.56
ER+PR+HER-	365 (79.7%)	231 (79.1%)	70 (82.4%)	64 (79.0%)	
ER+PR-HER-	55 (12.0%)	33(11.3%)	11(12.9%)	11 (13.6%)	
ER-PR-HER-	10(2.2%)	9 (3.1%)	1(1.2%)	0 (0%)	
HER2+	28 (6.1%)	19 (6.5%)	3 (3.5%)	6 (7.4%)	
Tumor multifocality presents^g^	148 (30.7%)	98 (30.9%)	21 (24.4%)	29 (25.8%)	0.32
Positive margin rate^h^	186(38.3%)	132(41.4%)	28 (32.9%)	26 (31.7%)	0.15
Follow-up time (years)^a^	8.0 (6.5)	9.9 (6.9)	4.1 (3.5)	4.6 (3.6)	< 0.001

*BCS* breast-conserving surgery; *ORM* oncoplastic reduction mammoplasty; *ER* estrogen receptor; *PR* progesterone receptor; *HER2* human epidermal growth factor receptor 2

^a^Data available in 494 cases

^b^Data available in 489 cases

^c^Data available for 368 cases

^d^Data available for 463 cases

^e^Data available in 483 cases

^f^Data available in 453 cases

^g^Data available in 482 cases

^h^Data available in 486 cases

Overall, 66 % (*n* = 326) of the patients underwent lumpectomy alone (standard BCS), 17.4 % (*n* = 86) underwent lumpectomy with immediate oncoplastic closure, and 16.6 % (*n* = 82) underwent immediate ORM. The rate of BCS as the initial surgery did not change over time, but the use of standard BCS without oncoplastic approaches was significantly less common in recent years. Before the year 2014, standard BCS was used in 86.7 % of cases compared with 39.4 % after 2014 (*p* < 0.001). The patients who underwent immediate ORM were significantly younger and more likely to have T3 tumors than those undergoing lumpectomy alone or lumpectomy with oncoplastic closure (average age, 58.1 vs 62.5 and 60.5 years respectively [*p* < 0.001]; T3 tumors in 24.4 % vs 8.2 % and 8.7 % respectively [*p* < 0.001]).

In the 368 BCS cases with resection volume data available, those who underwent oncoplastic surgery had significantly larger tissue volume excised (mean resection volume of 61.7 cm^3^ in standard BCS, 86.9 cm^3^ in lumpectomy with oncoplastic closure, and 189.5 cm^3^ in ORM; *p* < 0.01). Shave margin data were available for 463 patients, and of those, shave margins were obtained for 61.6 %. Use of shave margins was more common for those who also had oncoplastic surgery (53.0 % for standard BCS, 71.8 % for lumpectomy with oncoplastic closure, and 81.7 % for ORM; *p* < 0.01). There was no difference in tumor receptor subtype, grade, or presence of tumor multifocality by type of BCS. The mean follow-up time in the study cohort was 8 ± 6.5 years, with a significantly longer follow-up time for the patients undergoing lumpectomy alone (standard BCS) without oncoplastic techniques having a (Table 1).

### Positive Margins by Type of Surgery

Margin data were available for 486 (98.4 %) of the 494 patients with ILC who underwent BCS with or without immediate oncoplastic surgery, Of these, 186 patients (38.3 %) had positive margins at initial surgical resection. The univariate analysis showed no difference in positive margin rate by type of surgery performed (41.4 % for lumpectomy alone, 32.9 % for lumpectomy with oncoplastic closure, and 31.7 % for ORM). However, because type of BCS was associated with patient age, tumor size, and shave margin use, we evaluated a logistic regression model with adjustment for these factors. In this model, ORM was associated with significantly lower odds of positive margins than lumpectomy alone (odds ratio [OR], 0.34; 95 % CI, 0.17–0.66; *p* = 0.002).

Additionally, use of shave margins also was associated with significantly lower odds of a positive margin (OR, 0.48; 95 % CI, 0.30–0.75; *p* = 0.001). Larger tumor size was associated with an increased risk of positive margins (OR, 1.72 for every 1-cm increase in tumor size; 95 % CI, 1.50–2.0; *p* < 0.001), whereas older age was significantly associated with lower odds of positive margins (OR, 0.97 for every 1-year increase in age; 95 % CI, 0.95–0.98; *p* < 0.001) (Table 2).

**Table 2:** Multivariable logistic regression model for positive margins after breast-conserving surgery with adjustment for age, invasive lobular carcinoma (ILC) tumor size, and shave margins

	Odds of positive margins	
	Odds ratio (95% CI)	*P* value
BCS type		
Lumpectomy	Reference	Reference
Lumpectomy with oncoplastic closure	0.71 (0.39–1.29)	0.25
ORM	0.34 (0.17–0.66)	0.002
Age at diagnosis (years)	0.97 (0.95–0.98)	< 0.001
ILC tumor size (cm) Shave	1.72 (1.50–2.0)	< 0.001
Margins	0.48 (0.30–0.75)	0.001

Total *n* = 460

*BCS* breast-conserving surgery; *ORM* oncoplastic reduction mammoplasty

Of the 26 patients with positive margins after ORM specifically, 16 (61.5 %) underwent re-excision, 5 (19.2 %) underwent completion mastectomy, and 5 (19.2 %) were missing subsequent surgery data. Of the 16 ORM patients with positive margins who underwent re-excision, 2 (12.5 %) underwent completion mastectomy as a third surgical intervention.

### Rates of Successful BCS by Type of Surgery

Among all 494 patients in the study, 394 (79.8 %) ultimately had successful BCS, whereas 100 (20.2 %) underwent mastectomy to achieve clear margins. In the univariate analysis, the rates of successful BCS differed by procedure type because the patients who underwent either lumpectomy with oncoplastic closure or ORM had significantly higher rates of successful BCS than those who underwent lumpectomy alone (94.2 % and 87.8 % vs 73.9 %, respectively; *p* < 0.001). This finding persisted in the multivariable logistic regression analysis when the analysis adjusted for tumor size and age at diagnosis. This analysis showed that lumpectomy with immediate oncoplastic closure (OR, 7.5; 95 % CI, 2.8–20.0; *p* < 0.001) and immediate ORM (OR, 5.7; 95 % CI, 2.5–12.9; *p* < 0.001) were again associated with significantly higher odds of successful BCS than lumpectomy alone. Additionally, older age had marginally higher odds for successful BCS (OR, 1.1; 95 % CI, 1.0–1.1; *p* < 0.001), whereas larger ILC tumor size was associated with lower odds for successful BCS (OR, 0.7; 85 % CI, 0.6–0.8; *p* < 0.001) (Table 3).

**Table 3:** Multivariable logistic regression model for successful breast-conserving surgery with adjustment for age and invasive lobular carcinoma (ILC) tumor size

	Odds of successful BCS	
	Odds ratio (95% CI)	*P* value
Age at diagnosis	1.1(1.0–1.1)	< 0.001
BCS type		
Lumpectomy	Reference	Reference
Lumpectomy with oncoplastic closure	7.5 (2.8–20.0)	< 0.001
ORM	5.7 (2.5–12.9)	< 0.001
LLC tumor size (cm)	0.7(0.6–0.8)	< 0.001

Total *n =* 489

*BCS* breast-conserving surgery; *ORM* oncoplastic reduction mammoplasty

Of the 241 patients who had successful BCS after standard lumpectomy, this was achieved with a single operation for 172 (71.3 %), two operations for 63 (26.1 %), and three operations for 6 (2.5 %). Three of the subsequent operations were delayed ORM, and the remaining operations were simple re-excisions. Of the 81 patients who had successful BCS after lumpectomy with immediate oncoplastic closure, this was achieved with a single operation for 60 (74.1 %), two operations for 18 (22.2 %), and three operations for 3 (3.7 %). All subsequent operations were re-excisions except for one patient who had delayed ORM. Finally, among the 72 patients who had successful BCS following immediate ORM, this was achieved with a single operation for 56 (77.8 %) and two operations for 16 (22.2 %, all re-excisions; notably, 2 of these re-excisions were performed for negative but close margins), with no patients having a third operation.

### Recurrence-Free Survival

**Fig. 1:**
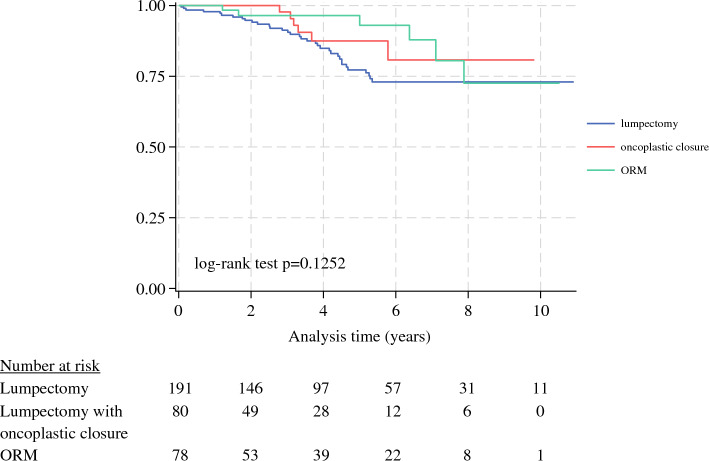
Kaplan–Meier recurrence-free survival curves based on the initial surgical procedure performed

During the study period, with a mean follow-up time of 8 years, 46 recurrence events (16 local and 30 distant) occurred. In the univariate analysis, RFS did not differ significantly between the patients who underwent lumpectomy with oncoplastic closure or immediate ORM and those who had standard lumpectomy (Fig. 1).

To assess the relationship further between type of surgery and RFS, a multivariable Cox proportional hazards model was developed and adjusted for age, tumor size, receptor subtype, and nodal status. The model demonstrated no differences in RFS estimates between standard lumpectomy alone and lumpectomy with immediate oncoplastic closure (HR, 0.81; 95 % CI, 0.33–2.0; *p* = 0.68) or immediate ORM (HR, 0.47; 95 % CI, 0.18–1.22; *p* = 0.12). Conversely, the factors associated with shorter RFS were presence of positive lymph nodes at surgery and ER+, PR–, HER2–, or HER2+ receptor subtypes compared with the ER+, PR+, HER2– subtype (Table 4).

**Table 4:** Cox proportional hazards model for recurrence-free survival after breast-conserving surgery with adjusment for age, tumor size, receptor subtype, and node status

	Recurrence-free survival	
	Hazard ratio (95% CI)	*P* value
BCS type		
Lumpectomy	Reference	Reference
Lumpectomy with oncoplastic closure	0.71 (0.33–2.0)	0.65
ORM	0.47 (0.18–1.22)	0.12
Age at diagnosis (years)	1.01 (0.98–1.04)	0.55
ILC tumor size (cm)	1.03 (0.86–1.23)	0.75
Tumor receptor subtype		
ER+PR+HER-	Reference	Reference
ER+PR-HER-	3.49(1.7–7.15)	0.001
ER-PR-HER-	2.59 (0.71–9.39)	0.015
HER2+	2.79 (0.81–9.58)	0.104
Node positivity	1.12(1.06–1.17)	< 0.001

Total *n* = 331

*BCS* breast-conserving surgery; *ORM* oncoplastic reduction mammoplasty; *ER* estrogen receptor; *PR* progesterone receptor; *HER2* human epidermal growth factor receptor 2

## Discussion

In this cohort of 494 patients with ILC, we found a significant benefit of immediate oncoplastic surgery over lumpectomy alone, as evidenced by a significantly lower risk of positive margins and higher rates of successful BCS. Notably, this cohort included a relatively high proportion of patients who would typically not be considered candidates for BCS, with 11.1 % overall having T3 tumors and 24.4 % of the ORM group having T3 tumors. Such cases have been described as “extreme oncoplasty” and likely contribute to the overall high positive margin rate seen in this study.^29^

Whereas prior analyses of oncoplastic surgery report positive margin rates ranging from 6.2 to 27.8%, our study showed that 38.3 % of patients experienced positive margins at initial resection. This may reflect the higher risk for positive margins in ILC cases generally, as several other investigators have pointed out.^30–32^ Notably, a prior study of extreme oncoplastic surgery, not stratified by histologic subtype, reported a positive margin rate of 54.5 %, reflecting the higher baseline risk with these larger tumors. Despite this, there was no increased risk of recurrence events for these patients with ILC who underwent immediate oncoplastic surgery.

These findings are quite consistent with several other series showing the oncologic safety of oncoplastic surgery for patients with breast cancer in general, but our results are the first to focus on ILC specifically, a tumor type for which surgical outcomes are known to differ from those for IDC.^13,25,26^

Prior investigators have raised concerns about the oncologic safety of immediate oncoplastic surgery for patients who have a high risk of positive margins, with some citing the technical challenges of re-excision after oncoplastic surgery as a potential barrier to its implementation.^33^ In our study, approximately 20 % of the patients who had immediate oncoplastic surgery also underwent re-excision, with no long-term detrimental effect seen on recurrence rates by surgery type. These data suggest that re-excision after oncoplastic surgery for ILC is indeed feasible and safe. Although we did not evaluate surgical complications and aesthetic outcomes, other investigators have shown no ill effect of re-excision on these outcomes after oncoplastic surgery.^30^

Importantly, the majority of the patients in the oncoplastic group were able to achieve successful BCS (94.2 % of the lumpectomy with oncoplastic closure group and 87.8 % of the ORM group), and most of these (>70 %) did so with a single operation. Notably, others have shown similar rates of successful BCS with the use of oncoplastic surgery, ranging from 90 to 94% in populations that were not restricted to ILC.^30,32–34^ We attribute our slightly lower rates of successful BCS in the ORM group to the diffuse growth pattern in ILC and the relatively high proportion of patients with T3 tumors in this cohort. We found significantly larger tissue volume of excision in those undergoing immediate oncoplastic surgery, and this more extensive surgical resection at the initial operation likely increases the likelihood of negative margins for diffusely growing tumors such as ILC.

One limitation of our study was the lack of data on breast size, as oncoplastic approaches may be more challenging for those with smaller breasts. However, recent investigators have described techniques that may be used even in the setting of less breast volume at baseline.^35^

Our findings suggest that oncoplastic surgery can be safely performed for those with ILC at the time of initial surgery despite the high positive margin risk for this tumor type. Indeed, immediate oncoplastic surgery can confer additional benefits beyond the improvement in negative margin rates. Reduction of ptosis can facilitate radiation planning and reduced skin dose.^36,37^ Additionally, for those who do have positive margins after the initial operation and opt for completion mastectomy, reduction in the size of the skin envelope with ORM first can facilitate nipple preservation at the time of subsequent mastectomy.^38–41^

Despite our relatively large cohort of ILC patients, the retrospective design and single-institution nature of this study presented inherent weaknesses. Additionally, the extended range of years during which patients underwent surgery for ILC (1995–2023) may have presented biases due to evolving surgical techniques, adjuvant therapies, and patient preferences over time, which could not be taken into account. Furthermore, our results should be taken in the context of our institutional approach, in which oncoplastic procedures routinely involve direct collaboration between breast and plastic surgery teams during both the initial resection and any necessary re-excisions. When re-excision is needed after oncoplastic surgery, careful review of preoperative imaging and multidisciplinary discussion between breast and plastic surgeons to review the oncoplastic approach (e.g., pedicle design) is pursued to improve accuracy of re-excision. Additionally, clips are routinely used to mark the lumpectomy cavity for radiation, which also may facilitate re-excision when necessary. Although data on the use of breast MRI in this cohort were not available, future analyses on whether this imaging method also helps in selecting appropriate candidates for immediate oncoplastic surgery and in guiding re-excisions would be of interest.

Overall, our findings provide evidence that immediate oncoplastic surgery can be considered for patients with ILC. Indeed, a recent analysis comparing immediate and delayed oncoplastic surgery among 39 patients with ILC found no differences in complications or high rates of successful BCS between the groups.^24^ In fact, it may be that patients with the highest risk of positive margins, such as those with ILC, stand to benefit the most from immediate oncoplastic surgery. This is supported by our findings, which show significant reductions in positive margin rates and completion mastectomy rates with immediate oncoplastic surgery versus standard lumpectomy in our cohort. Additionally, our finding of similar RFS by type of surgery performed further affirms the oncologic safety of immediate oncoplastic surgery even in the setting of diffusely growing tumors such as ILC. As such, our results support the use of immediate oncoplastic approaches for patients with ILC who desire BCS.

## Conclusions

Our analysis of 494 patients with ILC demonstrated the distinct role of immediate oncoplastic surgery in optimizing surgical outcomes for patients with high-risk tumor histology. At our institution, we found that immediate oncoplastic surgery was associated with significantly lower odds of positive margins, higher rates of successful BCS, and no negative impact on RFS compared with lumpectomy alone. These findings affirm the oncologic safety of immediate oncoplastic surgery even in the setting of diffusely growing tumors such as ILC. The results also support the notion that patients undergoing immediate ORM may experience improved surgical outcomes without increasing the risk of long-term recurrence.
